# Natural compounds ursolic acid and digoxin exhibit inhibitory activities to cancer cells in RORγ-dependent and -independent manner

**DOI:** 10.3389/fphar.2023.1146741

**Published:** 2023-04-26

**Authors:** Hongye Zou, Yatian Yang, Hong-Wu Chen

**Affiliations:** ^1^ Department of Biochemistry and Molecular Medicine, School of Medicine, University of California, Davis, Sacramento, CA, United States; ^2^ UC Davis Comprehensive Cancer Center, University of California, Davis, Sacramento, CA, United States; ^3^ VA Northern California Health Care System, Mather, CA, United States

**Keywords:** ursolic acid, digoxin, RAR-related orphan receptor gamma (RORγ), prostate cancer, breast cancer, context-dependent, natural products (NP)

## Abstract

Natural compounds ursolic acid (UA) and digoxin isolated from fruits and other plants display potent anti-cancer effects in preclinical studies. UA and digoxin have been at clinical trials for treatment of different cancers including prostate cancer, pancreatic cancer and breast cancer. However, they displayed limited benefit to patients. Currently, a poor understanding of their direct targets and mechanisms of action (MOA) severely hinders their further development. We previously identified nuclear receptor RORγ as a novel therapeutic target for castration-resistant prostate cancer (CRPC) and triple-negative breast cancer (TNBC) and demonstrated that tumor cell RORγ directly activates gene programs such as androgen receptor (AR) signaling and cholesterol metabolism. Previous studies also demonstrated that UA and digoxin are potential RORγt antagonists in modulating the functions of immune cells such as Th17 cells. Here we showed that UA displays a strong activity in inhibition of RORγ-dependent transactivation function in cancer cells, while digoxin exhibits no effect at clinically relevant concentrations. In prostate cancer cells, UA downregulates RORγ-stimulated AR expression and AR signaling, whereas digoxin upregulates AR signaling pathway. In TNBC cells, UA but not digoxin alters RORγ-controlled gene programs of cell proliferation, apoptosis and cholesterol biosynthesis. Together, our study reveals for the first-time that UA, but not digoxin, acts as a natural antagonist of RORγ in the cancer cells. Our finding that RORγ is a direct target of UA in cancer cells will help select patients with tumors that likely respond to UA treatment.

## 1 Introduction

Natural products isolated from plants or microorganisms are excellent sources for novel drug discovery (Lin et al., 2020; Atanasov et al., 2021). These naturally active products have relatively high oral bioavailability, special biological activities and known insights of safety and efficacy. Among the natural products, ursolic acid (UA) and digoxin have been reported to possess anti-cancer activity by disrupting multiple signaling pathways. UA is a pentacyclic triterpenoid presented in plants, fruits and herbs, including apple, basil and rosemary. It can inhibit NF-κB and STAT3 signaling (Shanmugam et al., 2011b; Shanmugam et al., 2012), and activate JNK-mediated apoptosis (Zhang et al., 2010b) in prostate cancer cells and tumors. UA also downregulates STAT3 (Sathya et al., 2014) and FoxM1 (Wang et al., 2012) signaling pathways in breast cancer cells. Digoxin can be isolated from foxglove plant. It potently inhibits the sodium potassium adenosine triphosphatase (Na+/K + ATPase) and is clinically used for heart diseases, including atrial fibrillation and heart failure. Recent studies suggest that digoxin can display anti-proliferation activity in cells of prostate cancers and breast cancers (Platz et al., 2011; Busonero et al., 2020). Both UA and digoxin have been evaluated in ongoing or completed clinical trials for treatment of several types of cancer. However, their direct targets in cancer cells and tumors remained unclear.

UA has been reported as an inhibitor of amyloid *β* interaction with its receptor CD36 (Wilkinson et al., 2011). Largely based on results from reporter gene assays, UA was also characterized as modulators of members of the nuclear receptor (NR) family of transcription factors, specifically as agonist of PPARα (Jia et al., 2011), and antagonist of LXRα (Lin et al., 2018) and RORγt, a T cell-specific isoform of RORγ (Xu et al., 2011). UA strongly reduces IL-17 expression in naïve CD4^+^ T cells and blocks the differentiation of T helper 17 (Th17) cells. Interestingly, UA does not appear to modulate the function of RORα, which is another member of the ROR subfamily of NR. In an early search for modulators of RORγt, digoxin was also shown to possess antagonistic activities to RORγt in Th17 cells (Huh et al., 2011). Thus, UA and digoxin are the two major natural compounds that were identified as modulators of RORγt in the early studies (Huh et al., 2011; Xu et al., 2011).

Recently, in search for alternative therapeutic targets for advanced cancer, RORγ in tumor cells was identified to play a critical role in tumor progression in certain types of cancer (Zou et al., 2022a), including castration-resistant prostate cancer (CRPC) (Wang et al., 2016; Wang et al., 2020; Zheng et al., 2020; Zhang et al., 2021), triple-negative breast cancer (TNBC) (Cai et al., 2019; Zou et al., 2022b), small cell lung carcinoma (SCLC) (Chen et al., 2022) and pancreatic ductal adenocarcinoma (PDAC) (Lytle et al., 2019). In CRPC tumors and cells, RORγ directly activates androgen receptor (AR) expression and AR signaling (Wang et al., 2016; Zheng et al., 2020; Zhang et al., 2021). In TNBC cells and tumors, RORγ acts as a master activator of tumor cholesterol biosynthesis program (Cai et al., 2019; Zou et al., 2022b). Pharmacological and genetic inhibition of RORγ strongly block prostate cancer (PCa) and TNBC cell growth and metastasis, suggesting that RORγ is a novel therapeutic target for cancer (Wang et al., 2016; Cai et al., 2019; Wang et al., 2020; Zheng et al., 2020; Zhang et al., 2021; Zou et al., 2022b).

Although a large number of synthetic, small-molecule modulators of RORγ/RORγt have been identified (Pandya et al., 2018; Zou et al., 2022a), few studies made a direct comparison of their activities. Recently, it was reported that structurally distinct, small-molecule modulators can display large differences in their activities in altering the function of RORγ/RORγt in control of its target gene expression (Zou et al., 2022b). Here we examined the activities of UA and digoxin in cells of PCa and TNBC where the function of RORγ is relatively defined (Wang et al., 2016; Cai et al., 2019). We found that UA but not digoxin disrupted the previously defined, RORγ-targeted gene programs. Our results demonstrate that UA, not digoxin, acts as a natural antagonist of RORγ in PCa and TNBC cells.

## 2 Materials and methods

### 2.1 Cell culture

C4-2B, 22RV1, LNCaP, PC3 and HCC70 cells were cultured in RPMI1640 (Corning) supplemented with 10% FBS. DU145 and MDA-MB-468 cells were cultured in DMEM (Corning) supplemented with 10% FBS. Cells were grown at 37 °C in 5% CO2 incubators. Cells were obtained from ATCC and were regularly tested being negative for *mycoplasma*.

### 2.2 Chemicals

XY018 (purity >99%) was synthesized by WuXi AppTec. Ursolic acid (purity >95%) and digoxin (purity >98%) were purchased from Cayman.

### 2.3 Cell viability, proliferation and colony formation

For cell viability, cells were seeded in 96-well plates at 1000–2000 cells per well in a total volume of 100 µl of media. After 4 days of incubation of compounds, Cell-Titer Glo reagents (Promega) were added, and luminescence was measured on Varioskan™ LUX multimode microplate reader (Thermo Scientific), according to the manufacturer’s instructions. All experimental points were set up in triplicate, and the entire experiments were repeated three times. The estimated *in vitro* IC_50_ values were calculated by using GraphPad Prism 9 software. For cell proliferation, cells were seeded in 6-well plates at 2 × 10^5^ per well and treated as indicated. Total viable cell numbers were counted using Countess™ II Automated Cell Counter (Invitrogen). For colony formation assay, 500 cells were seeded in a well of 6-well plate and cultured for 21 days with the medium changing every 5 days. When the cell clone grew visible, the medium was removed, and the cells were fixed with 10% formalin for 10 min. The plated were washed with PBS for two times, and cell colonies were stained with 0.2% crystal violet (in 10% formalin) for 30 min. The above assays were performed in duplicates, and the entire experiments were repeated three times.

### 2.4 Luciferase reporter gene assay and plasmid transfection

Transient transfection and reporter-gene assays were performed as previously described with modification (Zou et al., 2022b). Briefly, cells were co-transfected with pLX304-RORγ or empty vector and 7 X RORE reporter plasmid using lipofectamine 3,000 (Cat. L3000015, Invitrogen). Renila plasmid was co-transfected for normalization. After 12 h of incubation, cells were treated with vehicle or different compounds as indicated for another 24 h. The luciferase activity was analyzed using Dual-Glo Luciferase Assay System (Promega) on Varioskan™ LUX multimode microplate reader (Thermo Scientific), according to the manufacturer’s instructions. All transfections were performed at least in triplicate, and each experiment was repeated three times.

### 2.5 qRT- PCR and western blotting analysis

Total RNA was isolated from cells using TRIzol™ Reagent (Cat. 15596018, Invitrogen). The cDNA was prepared using qScript™ cDNA SuperMix (Cat. 95048-100, QuantaBio). Quantitative PCR were performed as previously described with modification (Yang et al., 2012). Briefly, cDNAs were mixed with SYBR Green qPCR master mix (Cat. A25742, Applied Biosystems) and gene specific primers. The PCR were performed using the CFX96 Real-Time PCR Detection System (Bio-Rad). The fluorescent values were collected, and fold difference was calculated. *GAPDH* was used as the internal reference to normalize the relative level of each transcript. The experiments were performed at least three times. Primers are listed in Supplementary Table S1.

After cells were lysed, protein concentrations were measured and adjusted using DC™ Protein Assay Kit II (Cat. 5000112, Bio-Rad). Proteins were separated by SDS-PAGE gel and transferred onto PVDF membranes (Cat. IPVH00010, MilliporeSigma). Membranes were incubated with indicated primary antibodies at 4°C overnight and then subjected to second antibody incubation. Antibody-recognized proteins were visualized using ChemiDoc™ MP imaging system (Bio-Rad) after incubation with HRP substrate (Cat. WBLUR0500, MilliporeSigma). Antibodies used are shown in Supplementary Table S2.

### 2.6 RNA-seq and bioinformatics analysis

HCC70 cells were treated as indicated before RNA extraction. RNA-seq libraries from 1 µg total RNA were prepared and validated as previously described (Yang et al., 2012). Sequencing was performed on an Illumina HiSeq 2000 Sequencer at BGI Tech (Hong Kong). The FASTQ-formatted sequence data were analyzed using a standard BWA-Bowtie-Cufflinks workflow. Briefly, sequence reads were aligned to the reference human genome assembly (hg38) with BWA and Bowtie software. Subsequently, the Cufflinks package (Trapnell et al., 2010). Was applied for transcript assembly and quantification gene expression. To avoid spurious fold levels due to low expression values, only subsets of genes that have expression value of RPKM (reads per kilobase per million mapped reads) or FPKM (fragments per kilobase of exon model per million mapped reads) above 1 for either the vehicle treated cell, or the compound treated cells are included. GSEA was performed using the Java desktop software (http://www.broadinstitute.org/gsea) as described previously (Subramanian et al., 2005). Genes were ranked according to the shrunken limma log_2_ fold changes and the GSEA tool was used in ‘pre-ranked’ mode with all default parameters. Previous reported AR-activity signature genes (Asangani et al., 2014) were used in the GSEA analysis.

### 2.7 ChIP-seq data analysis

ChIP-seq assay was performed as previously described (Cai et al., 2019).

Fastq files from the ChIP-seq were processed by the pipeline of AQUAS Transcription Factor and Histone (https://github.com/kundajelab/chipseq_pipeline). Briefly, sequencing tags were mapped against the *Homo sapiens* (human) reference genome (hg19) by using BWA 0.7.1551. Uniquely mapped tags after filtering and deduping were used for peak calling by model-based analysis for ChIP-Seq (MACS; 2.1.0) to identify regions of enrichment over background. Normalized genome-wide signal-coverage tracks from raw-read alignment files were built by MACS2, UCSC tools (bedGraphToBigWig/bedClip; http://hgdownload.cse.ucsc.edu/admin/exe/linux.x86_64/), and bedTools (https://github.com/arq5x/bedtools2). Visualization of the ChIP-seq signal at enriched genomic regions (avgprofile and heatmap) was achieved by using deepTools (https://deeptools.readthedocs.io/en/develop/index.html).

### 2.8 Statistical analysis

Cell culture-based experiments were performed three times or more, with assay points triplicated. The data are presented as mean values ±SD. Statistical analyses were performed by GraphPad Prism software 9.

## 3 Results

### 3.1 Ursolic acid (UA) and digoxin differs in effectiveness of killing cancer cells

**FIGURE 1:**
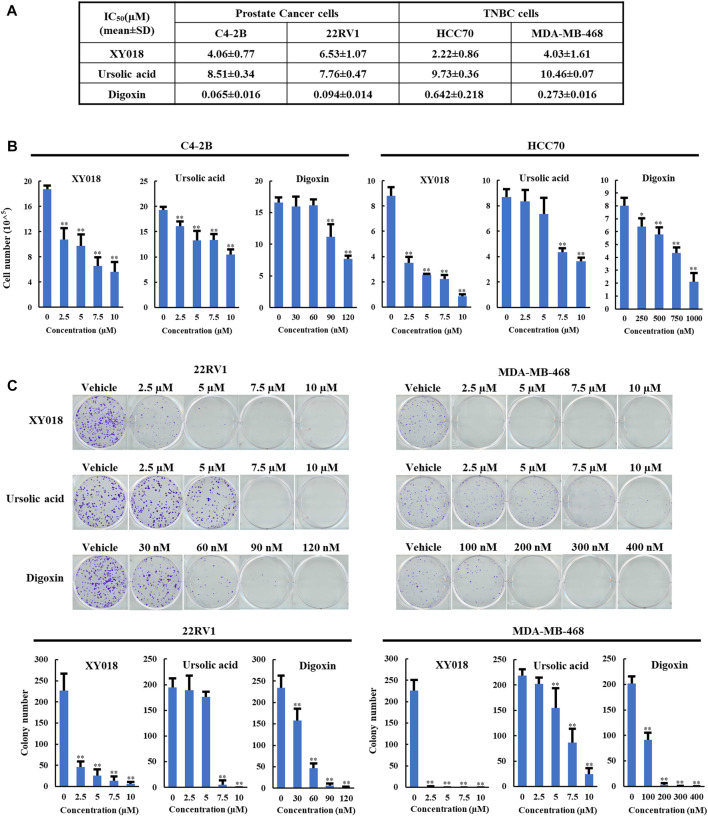
UA and digoxin display different effectiveness in inhibition of cancer cell growth when compared to synthetic RORγ antagonist XY018. **(A)**. The growth inhibition IC_50_ (μM) for synthetic RORγ antagonist XY018, natural RORγ antagonists ursolic acid and digoxin in indicated PCa and TNBC cell lines treated for 4 days. **(B)**. C4-2B and HCC70 cells were treated by different RORγ antagonists as indicated. Viable cells were counted after 4 days. Data are shown as mean ± SD. *n* = 3. Student’s t-test. *p < 0.05, **p < 0.01. **(C)**. 22RV1 and MDA-MB-468 cells were treated by different RORγ antagonists as indicated. Fourteen days later, representative images of colony formation were taken (top) and colonies were counted (bottom). Data are shown as mean ± SD. n ≥ 3. Student’s t-test. **p < 0.01.

To compare the anti-growth and -survival activities in cancer cells of UA and digoxin with synthetic RORγ inhibitors, we included XY018, which was characterized in its activity in antagonizing the function of RORγ in control of gene programs in the cancer cells and tumors (Wang et al., 2016; Cai et al., 2019; Chen et al., 2022). In the PCa and TNBC cells, UA displayed slightly weaker but comparable inhibitory activity in modulating cell growth and survival when compared to XY018. Specifically, XY018 displayed an IC_50_ of 2–6 µM in the two PCa cell lines (C4-2B and 22RV1) and the two TNBC cell lines (HCC70 and MDA-MB-468), while UA showed an IC_50_ of 7–10 µM for the same cell models. On the other hand, Digoxin displayed an IC_50_ in the sub-micromolar range for both PCa and TNBC cells with IC_50_ values over 50 fold lower than those of XY018 in the PCa cells (Figure 1A). Similar differences in their effectiveness were observed in assays of cell numeration and cell survival/colony formation assay (Figures 1B,C).

### 3.2 UA but not digoxin blocks transactivation activity of RORγ in cancer cells

**FIGURE 2:**
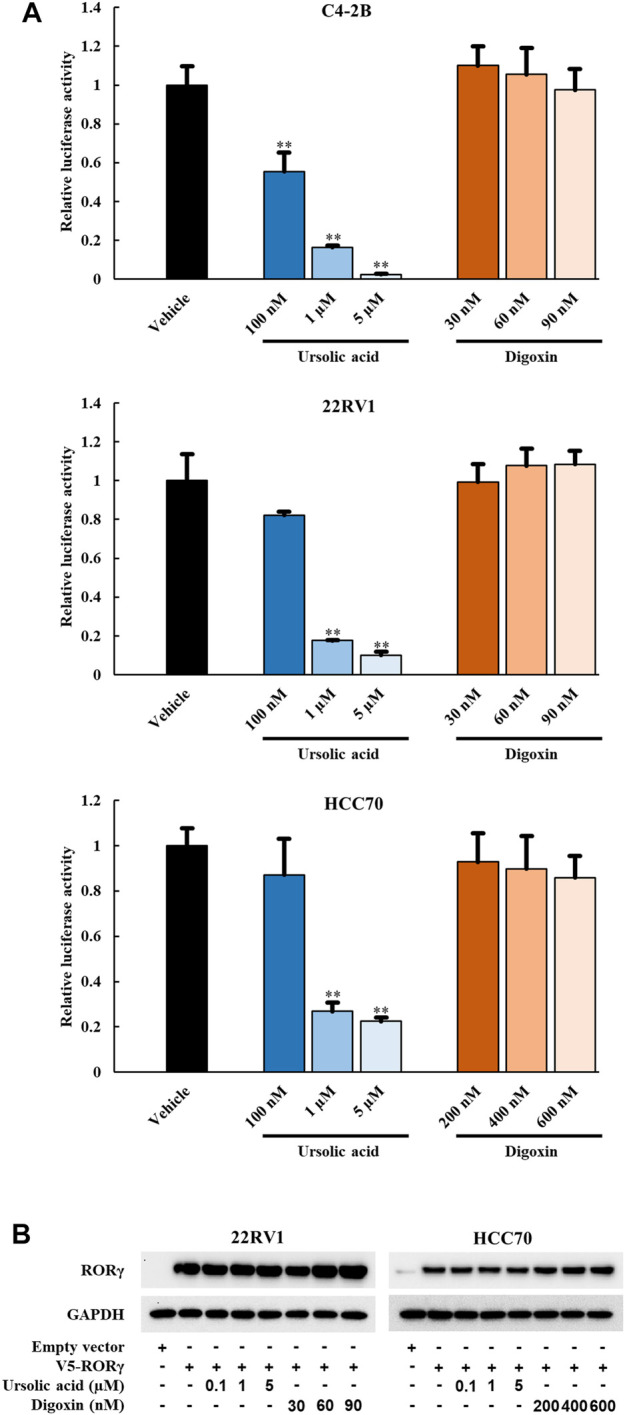
UA but not digoxin blocks transactivation function of RORγ in PCa and TNBC cells. **(A)** 7X-RORE luciferase reporter activity changes by treatment of ursolic acid or digoxin in C4-2B (top), 22RV1 (middle) or HCC70 (bottom) cells for 24 h. Normalized luciferase activity from cells treated with vehicle and transfected with RORγ-expressing plasmid were set as 1. Data are shown as mean ± SD. n ≥ 3. Student’s t-test. **p < 0.01. **(B)**. Immunoblotting of 22RV1 and HCC70 cells transfected with V5-RORC expression vector. Twelve hours after transfection, cells were treated with UA or digoxin at indicated concentration for another 24 h.

To examine whether the anti-growth effects of UA and digoxin is associated with their inhibition of RORγ functions in cancer cells, we performed luciferase reporter assay in different cancer cells. In PCa (C4-2B and 22Rv1) and TNBC cells (HCC70), UA diminished the RORγ-dependent activation of the reporter in a concentration-dependent manner (Figure 2A). Specifically, more than 70% inhibition of its transactivation was observed when cells were treated with 1 µM UA. However, no significant inhibitory effect was observed when cells were treated with digoxin (Figure 2A) at concentrations that display potent cell growth inhibition as shown in Figure 1. Additionally, the protein expression of RORγ remained unchanged when cells were treated with UA or digoxin (Figure 2B). Together, these data suggest that UA and digoxin may inhibit cancer cell growth through RORγ-dependent and -independent mechanisms.

### 3.3 UA but not digoxin disrupts RORγ-mediated AR expression and AR signaling

**FIGURE 3:**
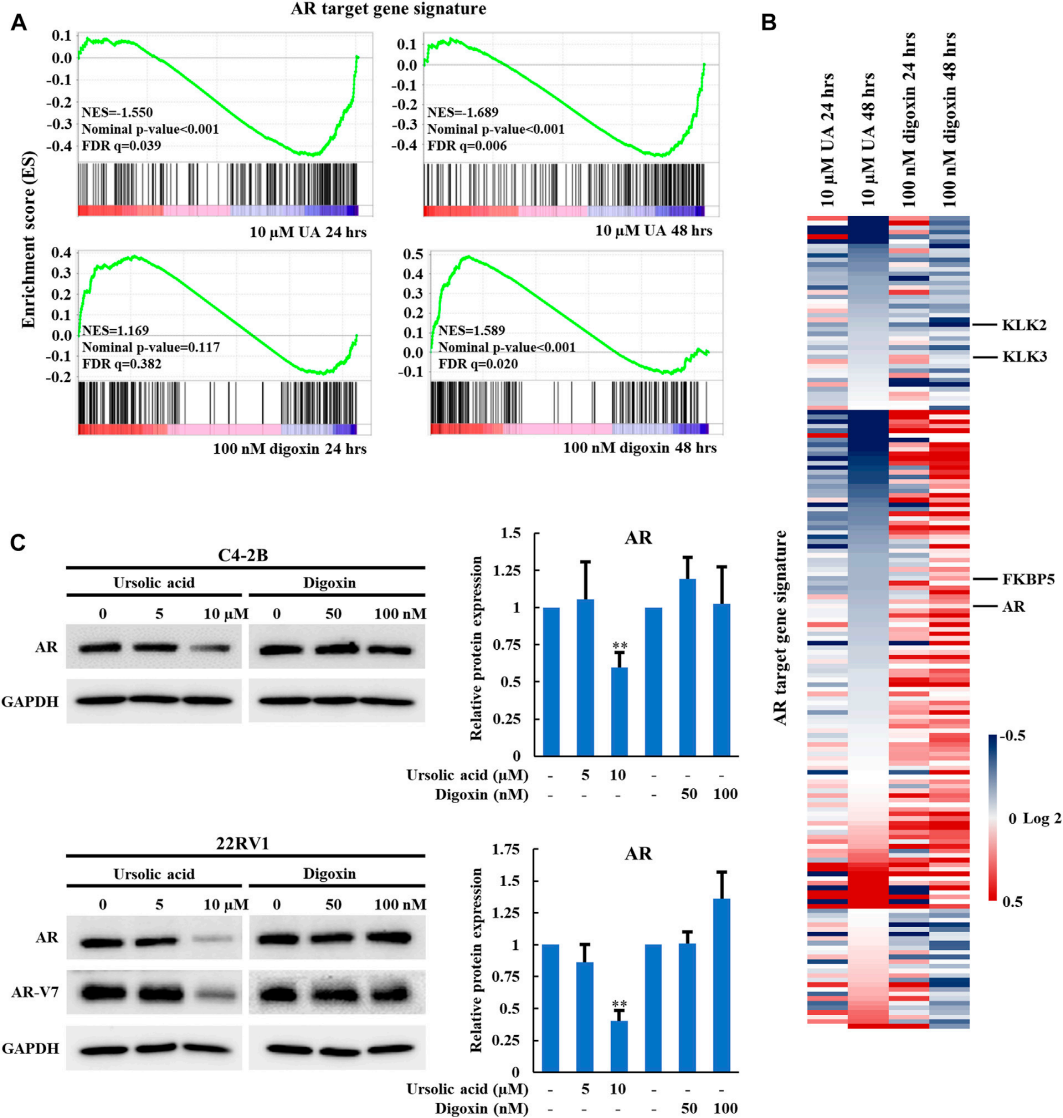
UA but not digoxin inhibits AR expression and AR-signaling in PCa cells. **(A)**. GSEA of the AR signaling pathway in C4-2B cell treated with UA (10 µM) or digoxin (100 nM) for 24 or 48 h. NES, normalized enrichment score. FDR, false-discovery rate. **(B)**. Heat map display of fold changes (in log2) of AR-signature gene mRNA analyzed by RNA-seq in C4-2B cell treated with UA (10 µM) or digoxin (100 nM) for 24 or 48 h. **(C)**. Immunoblotting of AR (full length) in C4-2B cell, AR (full length) and AR-V7 in 22RV1 cell treated with UA or digoxin at indicated concentration for 48 h (left). Quantification of Western blotting (right). AR expressions were normalized to that of GAPDH. Data are shown as mean ± SD. *n* ≥ 3. Student’s t-test. **p < 0.01.

In our previous studies, we demonstrated that RORγ directly activates AR gene expression and that synthetic RORγ antagonists reduce the expression of AR and its variant AR-V7 and AR-controlled gene programs in PCa cell lines and tumors (Wang et al., 2016; Zhang et al., 2021). To further examine whether the anti-growth effect of UA in PCa is through RORγ, we performed RNA-seq analysis of C4-2B cells treated by 10 µM UA. Gene-set enrichment analysis (GSEA) showed that an AR target gene signature gene set (Asangani et al., 2014) was significantly disrupted by UA treatment at 24 and 48 h (Figure 3A, top panels). In contrast, 48 h of digoxin treatment significantly enhanced the expression of the AR target gene signature (Figure 3A bottom right panel. Indeed, although some of the previously classified androgen-induced genes such as *KLK2* and *KLK3* (Asangani et al., 2014) were inhibited by both UA and digoxin treatment, *AR* and other AR-regulated genes like *FKBP5* were downregulated by UA while upregulated by digoxin treatment (Figure 3B). Consistent with RNA-seq analysis, UA reduced protein expressions of AR and its variant AR-V7 in C4-2B and 22RV1 cells. Conversely, digoxin treatment had no effects or slightly increased AR expression in C4-2B or 22RV1 cells, respectively (Figure 3C).

### 3.4 UA displays potent anti-proliferation activity in AR-positive but not AR-negative PCa cells

**FIGURE 4:**
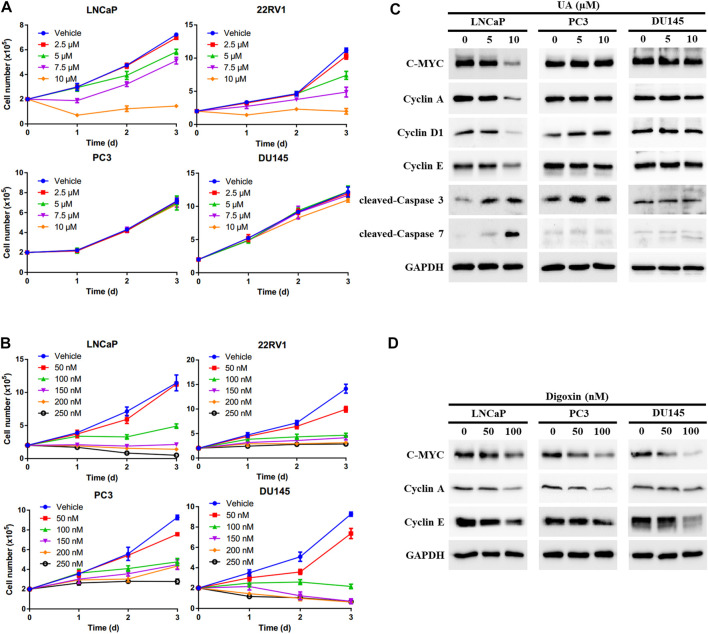
UA exhibits stronger inhibitory activity in AR-positive PCa compared with AR-negative PCa. (**A, B)** AR-positive PCa cells (LNCaP and 22RV1) and AR-negative PCa cells (PC3 and DU145) were treated by UA **(A)** or digoxin **(B)** at indicated concentrations. One, two and 3 days later, viable cells were counted. (**C, D)** AR-positive PCa cell (LNCaP) and AR-negative PCa cells (PC3 and DU145) were treated by UA **(A)** or digoxin **(B)** at indicated concentrations. Two days later, cells were harvested for Western blotting analysis of indicated proteins. Representative blots, *n* = 3.

To further examine whether UA inhibits PCa cell growth through disrupting AR signaling, we compared the anti-cancer effects of UA and digoxin in AR-positive and -negative PCa cells. As expected, UA showed significant stronger inhibitory activity in AR-positive PCa cells compared to AR-negative PCa. Specifically, 5 and 7.5 µM of UA was sufficient to strongly inhibit AR-positive LNCaP and 22RV1 cell growth, while 10 µM of UA had little or no effect on AR-negative DU145 and PC3 cell proliferation (Figure 4A). In contrast, digoxin displayed similar anti-growth effects in both AR-positive and AR-negative PCa cell lines (Figure 4B). In line with the cell growth effects, UA treatment potently reduced the protein expressions of key cell proliferation genes, including C-MYC, Cyclin A, Cyclin D1 and Cyclin E in LNCaP, but not in AR-negative PCa cell lines (PC3 and DU145). Additionally, expressions of critical cell apoptosis genes including cleaved Caspase-3 and -7 were induced by UA treatment in LNCaP cell and not in PC3 and DU145 cells. (Figure 4C). On the other hand, digoxin downregulated expressions of key cell cycle genes in all 3 cell lines tested (Figure 4D). Together, these data suggest that the anti-cancer PCa cell growth effects of UA is through its inhibition of RORγ function in control of AR-signaling pathway.

### 3.5 UA not digoxin alters RORγ-controlled expression of cell cycle and apoptosis genes

**FIGURE 5:**
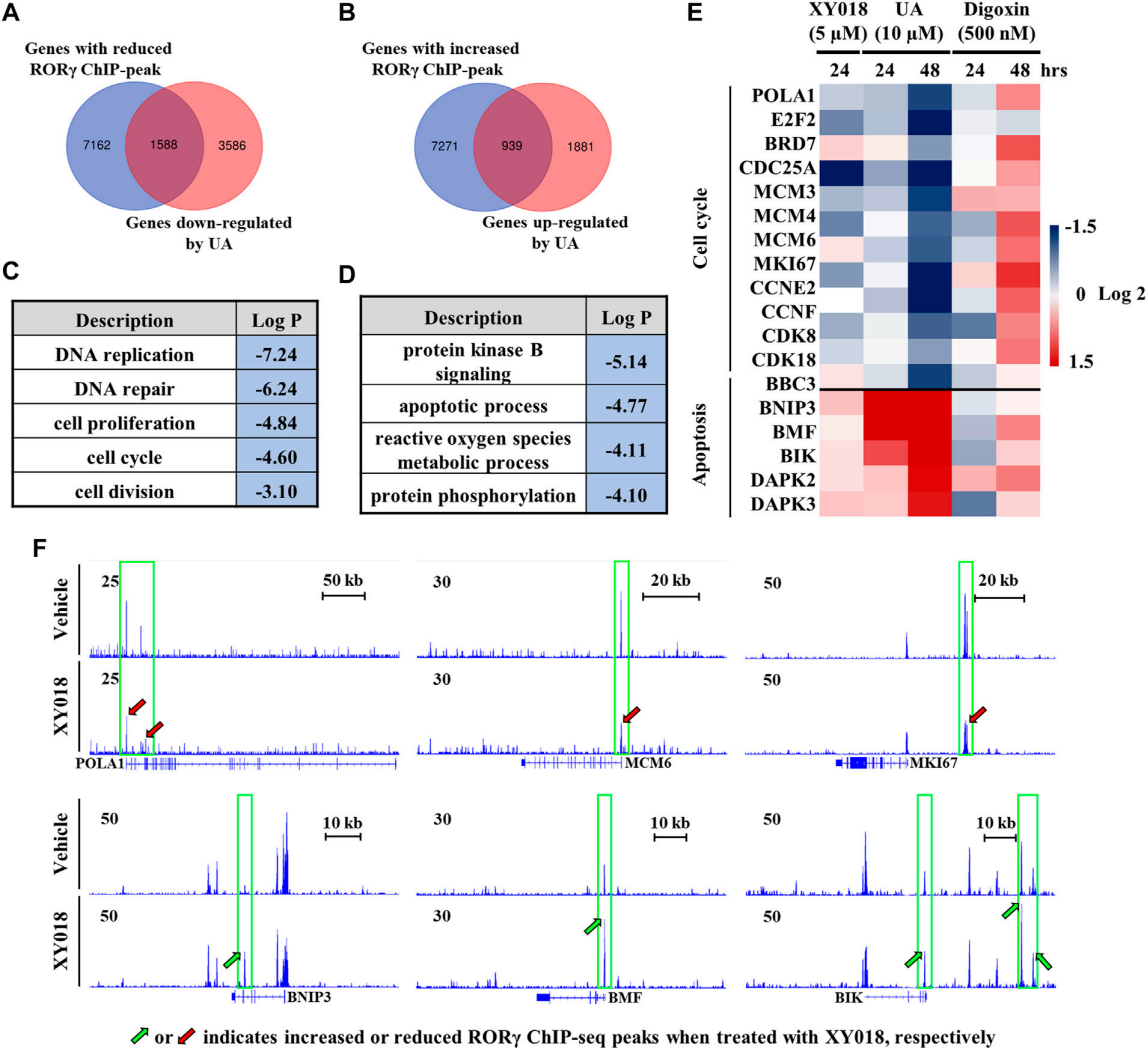
Expression of RORγ-controlled cell cycle and apoptosis genes was altered by UA but not digoxin in TNBC cells. (**A, B)** Venn diagram of number of genes with altered RORγ ChIP-peaks overlapped with genes altered by UA treatment. (**C, D)** Gene ontology analysis of genes with both reduced **(C)** or increased **(D)** expression and RORγ ChIP-peaks as shown in (**A)** or **(B, E)**. Heat map display of fold changes (in log 2) of RORγ direct target cell cycle and apoptosis gene mRNA in HCC70 cells analyzed by RNA-seq (UA) or qPCR (XY018 and digoxin) at indicated condition. **(F)**. ChIP-seq signal visualization of RORγ at representative cell cycle and apoptosis genes in HCC70 cells treated with 2.5 µM of XY018 or vehicle for 24 h.

To further elucidate the effects of UA on RORγ function in cancer cells, we analyzed RNA-seq data we obtained from TNBC cells treated by UA and our ChIP-seq data of RORγ genome occupancy in TNBC cells (Cai et al., 2019). Our analysis revealed a strong overlap between genes with altered expression by UA and genes that displayed RORγ ChIP-seq peaks. Specifically, 30.7% of genes downregulated by UA displayed reduced RORγ ChIP-seq peaks after the antagonist XY018 treatment (Figure 5A), whereas 33.3% of genes upregulated by UA had increased RORγ ChIP-seq peaks after XY018 treatment (Figure 5B). Gene ontology (GO) analysis of genes with both reduced expression and ChIP-seq peaks revealed that DNA replication and cell proliferation/division were among the most enriched programs with representative genes such as *POLA1, MCM6* and *MKI67* (Figures 5C,E, top panels). On the other hand, apoptotic process was one of the most enriched programs among genes being both UA-increased in expressions and XY018-increased in ChIP-seq peaks (e.g., *BNIP3, BMF* and *BIK*) (Figures 5D,E, bottom panels). Our further RNA-seq and qRT-PCR analyses showed that the mRNA expression of RORγ direct target genes involved in cell cycle/cell proliferation was reduced by both XY018 and UA, while genes involved in apoptosis were induced. In contrast, digoxin displayed either little or no effect at 24 h, or mostly activating effects at 48 h on those genes particularly those of the cell cycle/proliferation (Figure 5F). Together, the results suggest that like antagonist XY018, UA alters the expression of genes that are direct targets of RORγ whereas the effects of digoxin on gene expression in TNBC cells do not support the notion that digoxin acts through RORγ.

### 3.6 UA not digoxin suppresses RORγ-mediated cholesterol biosynthesis gene program

**FIGURE 6:**
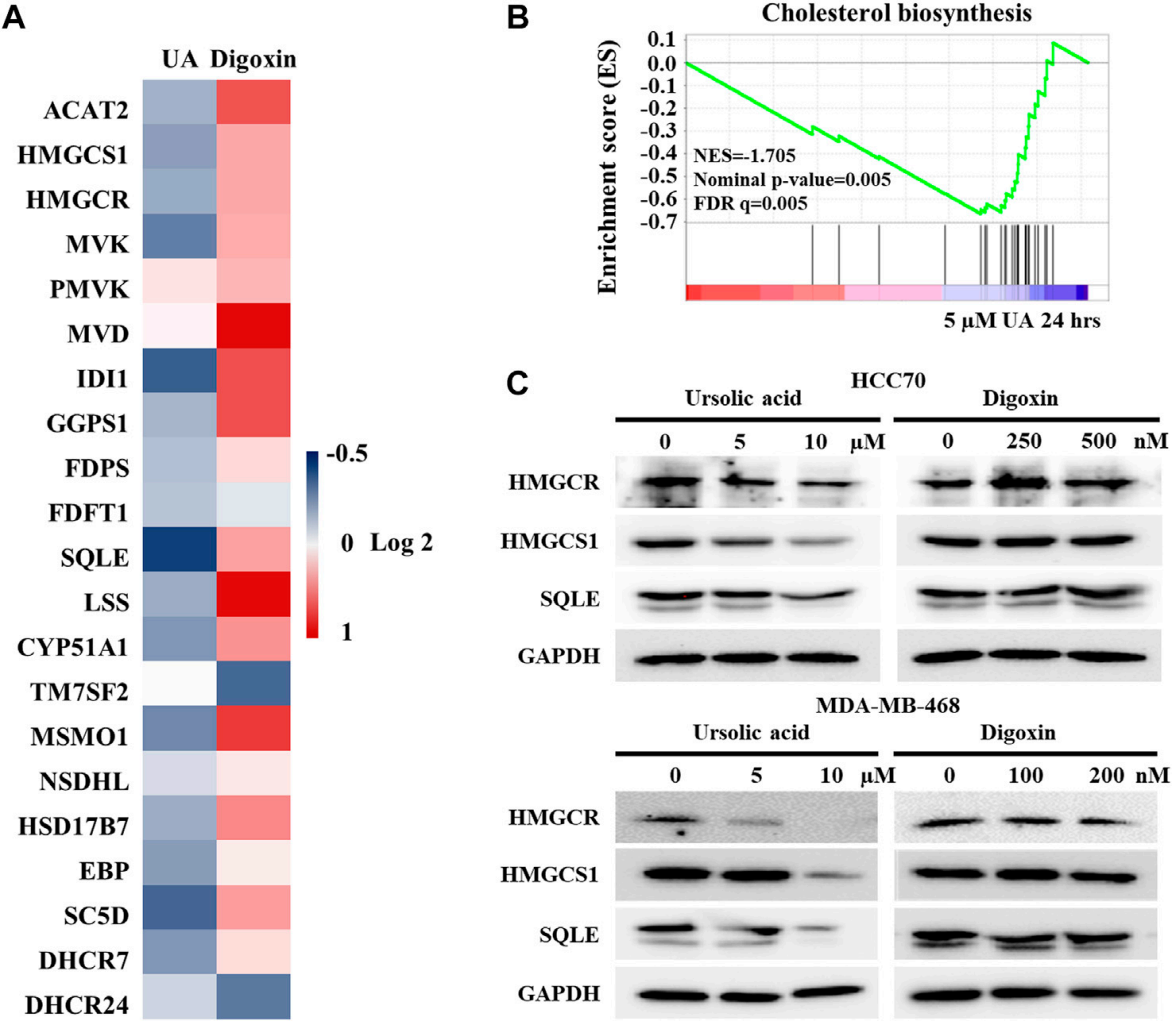
Expressions of cholesterol biosynthesis genes were altered by ursolic acid in TNBC cells. **(A)**. Heat map display of fold changes (in log 2) in mRNA expression of 21 cholesterol-biosynthesis genes in HCC70 cells treated by 5 µM UA (RNA-seq) or 250 nM digoxin (qPCR) for 24 h. **(B)**. GSEA of genes involved in cholesterol-biosynthesis pathway in HCC70 cell treated with 5 µM UA for 24 h. NES, normalized enrichment score. FDR, false-discovery rate. **(C)**. Immunoblotting of HMGCR, HMGCS1, SQLE and GAPDH in HCC70 and MDA-MB-468 cells treated with UA or digoxin at indicated concentration for 48 h. Representative blots, n = 3.

Our previous study demonstrated that RORγ directly controls cholesterol biosynthesis gene expression in TNBC cells (Cai et al., 2019). To further validate that UA but not digoxin targets RORγ-mediated signaling in TNBC, we analyzed the effects of UA and digoxin on cholesterol biosynthesis gene expression in HCC70 cells. Our RNA-seq and qRT-PCR analysis showed that UA treatment downregulated the expression of the majority of cholesterol-biosynthesis genes including those of the rate-limiting or key enzymes such as HMGCS1, HMGCR, MVK and SQLE. In contrast, digoxin upregulated their expression (Figure 6A). Indeed, GSEA showed that cholesterol-biosynthesis gene programs were significantly disrupted by UA treatment after 24 h of treatment (Figure 6B). Western blotting analysis also confirmed that protein expression of some of the key cholesterol-biosynthesis enzymes such as HMGCS1, HMGCR and SQLE were potently inhibited by UA treatment while remained unchanged when treated with digoxin (Figure 6C). Together with the other data in this study (Figures 2, 5), these results strongly suggest that the anti-tumor effects of UA but not digoxin in TNBC is at least partially through its inhibition of RORγ-mediated signaling.

## 4 Discussion

Digoxin, also known as digitalis, is prescribed to treat heart conditions. Its well-known mechanism of action (MOA) is its inhibition of Na^+^/K^+^ ATPase in the myocardium (Ren et al., 2021). Recent studies also demonstrated that digoxin can modulate several cellular signaling pathways including NF-κB (Wang et al., 2017) or EGFR-STAT3 signaling (Lin et al., 2015). In an early search for RORγ ligands, digoxin was claimed as a natural RORγt inverse agonist/antagonist in Th17 immune cells (Huh et al., 2011). Several later studies also showed that 5–10 µM of digoxin can suppress RORγt-mediated Th17 differentiation and IL-17 production (Fujita-Sato et al., 2011; Xiao et al., 2014; Lee et al., 2015). However, results from our study using cancer cell models do not support the conclusion that digoxin, at concentrations that are comparable to its use as an anti-cancer agent, can act as RORγ antagonist. We demonstrated in PCa cells that, at sub-micromolar concentrations, digoxin has modest but significant activating effects on the expression of RORγ direct target gene AR and AR signaling genes. Likewise, in TNBC cells, the expression of cell cycle and cholesterol biosynthesis gene programs that are directly activated by RORγ are also induced by digoxin treatment. These results strongly argue against the notion that digoxin can act as an RORγ antagonist in cancer cells. In fact, our finding is consistent with a recent study showing that digoxin can act as an RORγ agonist and induce RORγt-dependent transcription at sub-micromolar concentrations in the cells examined (Karaś et al., 2019). However, considering that digoxin can target Na^+^/K^+^ ATPase (Ren et al., 2021) and regulate other pathways (Lin et al., 2015; Wang et al., 2017), further studies are needed to determine whether any of the effects of digoxin in the cancer cells is through RORγ or other pathways.

Unlike digoxin, in this study we found that UA strongly inhibits the expression of AR, a direct target of RORγ in PCa cells. In our RNA-seq analysis, we revealed that UA can inhibit the expression of AR signaling genes that are positively regulated by AR (Asangani et al., 2014), consistent with our previous finding that RORγ stimulates the AR signaling gene program. In addition, we found that the strong anti-proliferation effect of UA can be observed only in AR-positive PCa cells but not AR-negative cells. These data together strongly suggested that the effects by UA treatment on AR expression and signaling is likely through its inhibition of RORγ function in the PCa cells. Several studies showed that UA can inhibit cancer cell and tumor growth by interfering with cell cycle, proliferation, apoptosis, inflammation, angiogenesis, and metastasis (Iqbal et al., 2018; Kornel et al., 2022; Zafar et al., 2022). However, there has been no evidence showing that UA can affect AR expression and/or AR signaling, which is the key driver of PCa development and progression. Of note, inhibition of AR by UA can be observed as low as 10 µM of UA, while the effects by UA on the other processes were reported at much higher concentration (20–50 µM) of UA (Zhang et al., 2010a; Shanmugam et al., 2011a; Shanmugam et al., 2011b; Li et al., 2022). Thus, it is possible that in PCa cells, disruption of AR signaling through inhibition of RORγ is the primary MOA of UA.

UA has been shown to display anti-growth effects in cells of different cancers, including prostate cancer, breast cancer, lung cancer, colorectal cancer, and pancreatic cancer (Khwaza et al., 2020; Zafar et al., 2022). However, the direct target of UA in cancer cells remains unclear. Our study here demonstrated in PCa and TNBC cells that UA inhibits the expression of gene programs such as AR signaling and cholesterol biosynthesis that are directly controlled by RORγ. Our integrated analysis of ChIP-seq and ATAC-seq data also revealed that gene programs affected by UA correlate closely with the ones directly controlled by RORγ in the cancer cells. Together, these results strongly support the conclusion that in the cancer cells, UA can act as an antagonist to RORγ. Although previous studies showed that UA can act as RORγt inhibitor in immune cells such as Th17 cells (Xu et al., 2011; Baek et al., 2014), our study here provides for the first-time evidence that UA displays RORγ antagonism activity in cancer cells. Similar to UA, recent studies identified additional natural compounds such as elaiophylin (Zheng et al., 2020) and N-hydroxyapiosporamide (Chen et al., 2022) as RORγ antagonists. Despite their structural differences, these natural compounds share similar inhibitory effects on the gene programs controlled by RORγ in the cancer cells and tumors. Given that natural agents often possess effects on multiple cellular and molecular targets, it is critical that thorough investigations are performed to better understand their MOA in order to further develop them for effective clinical use.

## Data Availability

The raw data generated in the study has been deposited in the GEO database (https://www.ncbi.nlm.nih.gov/geo/), under accession number GSE230039.
