# Supplementary material for: Natural compounds ursolic acid and digoxin exhibit inhibitory activities to cancer cells in RORγ-dependent and -independent manner
Source: Front Pharmacol. 2023 Apr 26;14:1146741. doi: 10.3389/fphar.2023.1146741 (PMC10169565; doi:10.3389/fphar.2023.1146741)
Supplement: Supplementary file 1 [file Table1.DOCX]

**Supplementary Table S1.**

| Primers for human qRT-PCR | |
| --- | --- |
| GAPDH-F | GCAGGGATGATGTTCTG |
| GAPDH-R | GTATGACAACAGCCTCAA |
| POLA1-F | GCTTCACCTCTGACCTTTAC |
| POLA1-R | GTACAGGGACTTGTCAGAATAC |
| E2F2-F | CAGCTACTGCTACCTACTACA |
| E2F2-R | CATCCACTCTGATGCACTTC |
| BRD7-F | CTTGATGAGACCACCAGATTG |
| BRD7-R | CGAACTCCATACGTGCTTAC |
| CDC25A-F | CTCCTCTCGTCATGAGAACTA |
| CDC25A-R | GGAAGATGCCAGGGATAAAG |
| MCM3-F | CCTATGCCAAGCAGTATGAG |
| MCM3-R | GGGTGGTGAGATCAGAATAAC |
| MCM4-F | CCTCTGGCTAAAGAAGAAGAAA |
| MCM4-R | CAGGGTAACGGTCAAAGAAG |
| MCM6-F | GGAAACACCTGATGTCAATCTA |
| MCM6-R | CCTCTTCTTCTTCCACCTTTC |
| MKI67-F | GTCACACCGAGGAATTAGTG |
| MKI67-R | CTTCACCTACTGATGGTTTAGG |
| CCNE2-F | CCTCCATTGAAGTGGTTAAGA |
| CCNE2-R | CCTCCAGCATAGCCAAATAG |
| CCNF-F | TCACCGGATTCTCCTATGAA |
| CCNF-R | CTGTCTTGTGTCACTCCTAATG |
| CDK8-F | CGGAAGAAGAACCTGATGAC |
| CDK8-R | GTGGATTGGAACGCTGATAG |
| CDK18-F | CCTATGCCACAGTCTTCAAA |
| CDK18-R | CACTGTCCAGGTACTCAAAC |
| BBC3-F | ATCCCATTGCATAGGTTTAGAG |
| BBC3-R | CTACAGCAGCGCATATACAG |
| BNIP3-F | CTCAGATTGGATATGGGATTGG |
| BNIP3-R | CAAATGAGAGAGCAGCAGAG |
| BMF-F | CAGTTTCCCAGCAGTCTTG |
| BMF-R | GTTCCTGTTCTCTTCTCCATTC |
| BIK-F | GGAGGACTTCGATTCTTTGG |
| BIK-R | TCTCCTTAAGTGTGGTGAAAC |
| DAPK2-F | GCTGACATGTGGAGCATAG |
| DAPK2-R | GTTTCCGGGTCTCTTTAACC |
| DAPK3-F | ATGCTGCTGGACAAGAAC |
| DAPK3-R | CTCAGGAGGATATAGGTGATGA |
| ACAT2-F | GTGCTGCAGCTGTCGTTCTTAT |
| ACAT2-R | CTTCCAGTGACCAACCTGCTTT |
| HMGCS1-F | CTTTCGTGGCTCACTCCCTTTC |
| HMGCS1-R | AGGGCAACAATTCCCACATCTT |
| HMGCR-F | GCACCAAGAAGACAGCCTGAATAG |
| HMGCR-R | TCTGAGGAGTCTGCATGGAAAGA |
| MVK-F | CCTCAGCTTACCCAACATTGGTATC |
| MVK-R | TCCGGCAGATGGACAGGTATAA |
| PMVK-F | AGTGGTTTCGGGAGGCCTAT |
| PMVK-R | TCAGGTTCTCCAACTGCTCCT |
| MVD-F | TGGCATCGGTGAACAACTTCC |
| MVD-R | CCCATCTGCCACTCCACAAAG |
| IDI1-F | CACACCCTGGATATGTGTTCTGTTT |
| IDI1-R | TCTGCAAGTGCTCCGGAAATG |
| GGPS1-F | GGCAGTTCCAAGCCAGTTTCTA |
| GGPS1-R | CCTCCCAAAGTGCTGGGATTAC |
| FDPS-F | GCCAAGGAAACAGGATGCTGATAG |
| FDPS-R | AGCTTCAGCAGGCGGTAGATA |
| FDFT1-F | GGTCCCGCTGTTACACAACTTT |
| FDFT1-R | GCCATCCCAATGCCCATTCT |
| SQLE-F | CCATGCTCCACTGACTGTTGTT |
| SQLE-R | AGATGAGAACTGGACTCGGGTTAG |
| LSS-F | GCGAGGAGCGGCGTTATTT |
| LSS-R | TGTAGGAGATGGCACAGGACTT |
| CYP51A1-F | TTGGCTGCCTTTGCCTAGTT |
| CYP51A1-R | GCTGCCCTGCCAAGAGTAAT |
| TM7SF2-F | CACCCTCACCGCTTTCATCTT |
| TM7SF2-R | CGGGTCGCAGTTCACAGAAATA |
| SC4MOL-F | CCTCCCAAAGTGCTGGGATTAC |
| SC4MOL-R | AAGTTCTTGGTGCCGGCTTT |
| NSDHL-F | GCCAGGAACGGCAAGATGAA |
| NSDHL-R | GCGAGACAGGAATGTCCAGAAAG |
| HSD17B7-F | CCACCACTGGCTTTGGAAGAA |
| HSD17B7-R | GCCTCCCAAAGTGCTGGAATTA |
| EBP-F | ACTGGCCTCAGCACCTAAGA |
| EBP-R | AACCCACACACTGCAAACCA |
| SC5D-F | GTTGCACCATCCCTGGTTTCT |
| SC5D-R | CTGCCCTCTGCAACTGATTTCT |
| DHCR7-F | CATTGACATCTGCCATGACC |
| DHCR7-R | ACAGGTCCTTCTGGTGGTTG |
| DHCR24-F | TGTTGCCTGAGCTTGATGAC |
| DHCR24-R | GACCAGGGTACGGCATAGAA |
