# Supplementary material for: Natural compounds ursolic acid and digoxin exhibit inhibitory activities to cancer cells in RORγ-dependent and -independent manner
Source: Front Pharmacol. 2023 Apr 26;14:1146741. doi: 10.3389/fphar.2023.1146741 (PMC10169565; doi:10.3389/fphar.2023.1146741)
Supplement: Supplementary file 2 [file Table2.DOCX]

**Supplementary Table S2.**

| Antibody | Vendor | Catalog number |
| --- | --- | --- |
| AR | Santa Cruz | SC-7305 |
| AR-V7 | Cell signaling | 68492 |
| Cleaved-Caspase 3 | Cell signaling | 9664 |
| Cleaved-Caspase 7 | Cell signaling | 9491 |
| C-MYC | Santa Cruz | SC-40 |
| Cyclin A | Santa Cruz | SC-271682 |
| Cyclin D1 | Santa Cruz | SC-8396 |
| Cyclin E | Santa Cruz | SC-247 |
| HMGCR | Santa Cruz | SC-271595 |
| HMGCS1 | Santa Cruz | SC-166763 |
| RORγ | Invitrogen | 14-6988-82 |
| SQLE | Santa Cruz | SC-271651 |
| GAPDH | Cell signaling | 2118 |
